# Abortion; Spontaneous and Induced*Read at meeting Central Texas Medical Association, Waco, Texas, January 10, 1900.

**Published:** 1900-03

**Authors:** J. A. Winfrey

**Affiliations:** Winnewood, I. T.


For Texas Medical Journal.

### Abortion; Spontaneous and Induced.*

BY J. A. WINFREY, M. D., WINNEWOOD, I. T.

I know it is in order to offer apologies for attempting to do justice to a subject of such importance as the one I have voluntarily selected; but if I can arouse sufficient interest to invite criticism, I shall feel that the time in preparing this paper has not been wasted or your patience imposed upon.

I shall speak of *abortion* as the premature expulsion of the fœtus before the time of viability.

The length of this paper will not permit me to go into detail, but to summarize a few leading facts relative to the different subjects that properly belong under one head.

In their natural order, I will mention some of the common causes of abortion, viz.: Over exertion, riding in jolting vehicles, or on horseback, tight lacing, or the weight of heavy skirts; dysentery, cystitis, constipation, inflammation or displacement of uterus or its appendages, laceration of cervix, malaria, syphilis, eruptive diseases, and diseases characterized by acute inflammation, intense mental excitement, and I'll not forget the " female regulators" so extensively advertised in the many "Ladies' Home" papers, and also, that little instrument *Uterine Sound* that should be put away with the trephine, to be used on *rare occasions.*

Diagnosis becomes a question with the first warning symptoms. Unfortunately these cases are seldom seen until the membranes rupture or hemorrhage occurs, and then it is not always possible to differentiate between a threatened abortion and one of metrorrhagia from other causes, as a mole or hydatid cyst; for a case is recalled where a patient was sure of pregnancy until the seventh month, when I removed a five pound hydatid.

The treatment is always preventive; for we are never justifiable in hastening the expulsion of the ovum until we are sure that structural changes have taken place in the ovum itself, or the placenta is completely detached.

A careful inquiry should be made for the cause or causes operating in the case, and it, or they removed. Absolute rest in bed, the lower bowels emptied, and the mind made tranquil with an opiate

---

*Read at meeting Central Texas Medical Association, Waco, Texas, January 10, 1900.

or sedative. A digital examination will decide what next to do. If displacements of womb or ovaries are found, they should, if possible, be corrected. Inflammatory conditions should be met with appropriate treatment. After all symptoms have subsided, constitutional conditions, as causative factors, should be met with constitutional remedies. Laceration of cervix should be left alone until after confinement, and then an operation advised.

Hemorrhage is always serious, considered from two standpoints—the safety of the mother, and the life of the child. The action of drugs is too slow and uncertain to be relied on, and it is here we can make use of the tampon, the great sheet-anchor in all such cases. The method of using the tampon is of no little importance, and if properly applied, not only insures the immediate safety of the patient from loss of blood, but gives the greatest comfort as well.

The size of the tampon should be just large enough to fill the vagina evenly and without undue pressure. The uterus must be placed in its normal position or else the trouble will be aggravated.

The patient should be placed in the knee-chest position to facilitate the correction of any displacement, and the tampon packed firmly around the cervix, care being taken that no pressure is made on the bladder. The material used is not always one of selection. A clean, white silk, or soft cotton handkerchief may be used in emergencies. Sterilized lamb's wool is the ideal tampon, because it retains its elasticity when wet, and the evenness with which it can be applied. It is also the most desirable, because it hastens coagulation quicker than any other dressing.

What to do when abortion is inevitable will be considered under the head of the after treatment of induced abortion.

Induced abortion is a question of no uncommon interest because of the social and legal questions involved.

The medical profession has never given the subject that attention that is necessary to a thorough understanding of its purely medical aspects; and I feel safe in saying that there are not a few who believe that it never becomes a necessary operation. There are many who will not assume the responsibility even when convinced of its necessity. Others, from false conceptions of what is right or wrong, will never advise it.

Those that would shirk the responsibility of motherhood without foregoing the pleasures of conjugal or illicit intercourse are many, and their importunities should be given consideration, for to whom but the physician can she go for advice. If she be a wife, make plain to her the enormity of the sin she contemplates, as well as the physical dangers to be encountered. If she is unprotected by conjugal ties, she deserves your special commiseration.

Who but the theological bigot would brand her publicly with the scarlet letter while the partner in her sin remains the lion of the social world? Do not tell her she has done wrong—this she too well knows. Remember she is human, and is probably the victim of a misplaced confidence.

Could the natural order of such conditions between man and woman be reversed, womankind would stand appalled at the social status of man, and our orphan asylums would cover the face of the earth. It is not the fulfillment of conception she dreads so much as social ostracism, and 'tis this that the physician should employ his tact in averting. She might be advised to take, ostensibly, a trip to some distant relative. Fortunately we have havens of "retreat" for such shipwrecks. Deprive her of compass or rudder and ere long no memorial is left of that poor mariner, save the sad sighing of the sea—a mournful requiem over the shipwrecked soul, as she sleeps deep down amid the sea weeds of life's ocean wave.

Induced abortion becomes a crime when done for purposes other than to save the life or reason of the mother.

The indications for interrupting the natural course of pregnancy in the early months are, first, mechanical obstruction, as deformity of pelvis and tumors. Second, nervous diseases, not amenable to treatment, as puerperal mania, chorea and neuralgia. Third, organic diseases of the heart, aggravated by the puerperal state. Fourth, pernicious vomiting of pregnancy. It is to pernicious vomiting that I wish to particularly call your attention, and to urge an early operation in all such cases. There are many cases of vomiting that need no surgical interference, while there are a few that do. It is the *few* that deserve special attention. When you stand by the bedside of some woman and see her die, vomiting, begging for relief that you are powerless to give, the question will then arise whether other agents besides drugs could have been employed and her life saved. Some of the most painful memories I have, and the darkest page in the history of my life, bears particularly on this part of the subject. I saw one woman die vomiting, and I said then I would let no other die from the same cause, and it has been my good fortune to make my promise good.

In my town, a young wife died after vomiting for three months—the attending physician had "conscientious scruples." I will mention a case from my own practice; a young wife with the following history: In 1898 she became pregnant, and vomited incessantly from the first month of conception till she was taken to Kansas City, where she was prematurely delivered at the seventh month of gestation in order to save her life. The child lived only a few hours and with difficulty was the life of the mother saved. She again became pregnant in October, 1899; vomiting began the second week and continued for three weeks, when she came under my observation. Knowing her previous history, and after employing the usual remedies without effect, I advised an immediate operation, which I did, and the patient was well within a week. These are object lessons in the great kindergarten school of medicine.

Question: When must we operate in such cases? When medicines fail to do good, and before the patient approaches the danger line from exhaustion. Some of you may say that you have never seen a case that was not relieved with this or with that remedy. To you, I say, you have been fortunate. I have tried every known drug as remedies for such cases, per rectum, stomach, or hypodermically; blistered, dilated the cervix, painted the same with cocaine, silver, iodine and carbolic acid, only to be disappointed in the end.

Before attempting the operation, the full consent of the patient and immediate relatives should be obtained, and you should state in language plain that you will not be responsible if the case should terminate unfavorably; and unless you can proceed with that understanding, withdraw from the case.

Before invading the uterus, strict antiseptic precautions should be observed, the lower bowels emptied, and the patient made comfortable in bed.

To facilitate the removal of the fœtal mass *en bloc,* care should be taken that the membranes are not ruptured. The Krause method is the one usually employed. The dangers to be guarded against in the use of the catheter are, first, entrance of air into uterus, and second, injury to the fundus by the resistance of the point of the catheter during uterine contraction. These dangers may be obviated by filling the end of the catheter with sterilized water before introducing, and regulating the depth it is to be introduced, which should not be more than two and a half or three inches, in the early months of pregnancy. The lumen of the catheter should be closed by tying at a point just outside the cervix, and a small safety pin passed through, or fixing a button at the same point. The catheter should be held in place with a tampon, and removed when uterine contraction commences, which will be within twenty-four or forty-eight hours. After an antiseptic douche, the tampon should be reapplied, and the case treated on the expectant plan until the uterus empties itself, or more radical measures are indicated.

What to do when portions of the placenta are retained is an undecided question. I believe it best to wait, unless hemorrhage is likely to endanger the life of the parent, or sepsis is feared, as indicated by a foul smelling discharge. The patient is usually exhausted from pain, loss of blood, or loss of sleep, and if possible, time should be given for her to recuperate before attempting instrumental or other forcible extraction of the retained membranes. After emptying the uterus, the slightest ill-smelling discharge calls for a free irrigation of uterus with hot sterilized water, and the entire cavity swabbed with campho-phenique, or iodized-phenol. No tampon should be used at this time, as it prevents free drainage and interferes with the necessary frequent antiseptic vaginal douches.

